# The modulation of actin dynamics via atypical Protein Kinase-C activated Cofilin regulates metastasis of colorectal cancer cells

**DOI:** 10.1080/19336918.2018.1546513

**Published:** 2018-11-18

**Authors:** S M Anisul Islam, Rekha Patel, Raja Reddy Bommareddy, Khandker Mohammad Khalid, Mildred Acevedo-Duncan

**Affiliations:** Department of Chemistry, University of South Florida, Tampa, FL, USA

**Keywords:** CRC, metastasis, actin dynamics, PKC-ι, PKC-ζ, cofilin

## Abstract

Colorectal cancer (CRC) is the third most common cancer in the United States. The exact mechanism of CRC cells metastasis is poorly understood. Actin polymerization is thought to be an initial step in the cancer cell motility cycle which drives the formation of cell protrusions and defines the direction of migration. Cofilin, a significant actin-regulating molecule, regulates the migration of cancer cells by the formation of lamellipodia and filopodia, however, little is known about the upstream regulation of cofilin. In this study, the effect of atypical Protein Kinase C (atypical PKC) on Cofilin activity in CRC was studied. This study demonstrates that the atypical PKC inhibition impedes the metastasis of CRC cells by increasing phospho-Cofilin (S3) and changing actin organization.

## Introduction

Colorectal cancer (CRC) is one of the most common cancers of the gastrointestinal tract. The current therapeutic options for treating CRC are confined to surgical removal of the tumor, chemotherapy, and radiation. Metastasis is an essential factor in a patient’s death with CRC [1]. The assembly of actin filaments at the leading edge drives the movement of the cells by generating cell protrusion force during invasion and migration [2]. Actin-binding proteins (ABP) regulate actin filaments dynamics by controlling polymerization and depolymerization and the formation of actin-based bundles and cellular protrusions [3,4]. A significant member of ABP superfamily known as Cofilin/actin depolymerizing factor (ADF) plays an essential role in actin-filaments turnover by depolymerizing and severing filamentous actin [5–7]. The regulation of Cofilin activity depends on its phosphorylation and dephosphorylation [8]. The phosphorylation of Cofilin at Serine-3 by different kinases such as LIM kinase 1 (LIMK1) and LIM kinase 2 (LIMK2) make it unable to bind and sever filamentous actin [9,10]. However, phosphatases such as Slingshot (SSH), Protein Phosphatase1 (PP1), Protein Phosphatase 2A (PP2A), Protein Phosphatase 2B (PP2B), and chronophin dephosphorylate Cofilin at Serine 3 to render it active [11–13].

Although the exact mechanism of migration and chemotaxis is complex and far from complete elucidation, the Protein Kinase C (PKC) family of enzyme drew significant attention over the last few decades as a critical regulator of chemotactic cell migration [14,15]. PKC is a large family of phospholipid-dependent Serine/Threonine kinases activated by different extracellular molecules [16]. Based on the domain structures and regulation, there are three classes of PKCs: (1) Classic PKC, include PKC-α, βI, βII and γ, contain a phospholipid binding domain (C1) and a Ca^2+^ binding domain (C2), hence, are regulated by Ca^2+^ and lipid secondary messengers such as diacylglycerol; (2) Novel PKC, include PKC-δ, η, θ, and ϵ, contain phospholipid binding domain (C1) but lack Ca^2+^ binding domain, thus, regulated by lipid but insensitive to Ca^2+^; and (3) Atypical PKC, include PKC-ι, and PKC-ζ, neither have lipid binding domain nor Ca^2+^ binding domain, therefore insensitive to both diacylglycerol and Ca^2+^ [17].

Phosphorylation of many downstream substrates by PKC regulates the growth, transcriptional regulation, learning, memory, polarity, chemotaxis, migration, and adhesion in different cell types [18–20]. For instances, PKC-α mediated phosphorylation of integrin α6β4 drove the mobilization of integrin from hemidesmosomes and associated with actin cell protrusion to regulate the migration of cancerous cells [21]. In MDA-MB-468 breast cancer cells, PKC-δ plays an essential role in the persistent migration of EGFR overexpressed cells by controlling the EGF dependent phosphorylation of the light chain of myosin [22]. PKC-ι controls cell cycle progression by regulating CAK/CDK7 in glioma cells [23]. Moreover, PKC-ι is also involved in the metastasis and angiogenesis of pancreatic cancer via stimulating Rac1/MEK/ERK pathway [24]. Likewise, PKC-ζ regulates the survival, growth, and polarity of the cancer cells by regulating NF-ĸb and Jak/Stat signaling pathway [25]. Additionally, PKC-ζ converges with the Receptor Tyrosine Kinase (RTK), and G-protein coupled receptor-mediated chemotactic signals in breast and lung cancers cell lines [26,27].

**Figure 1.**
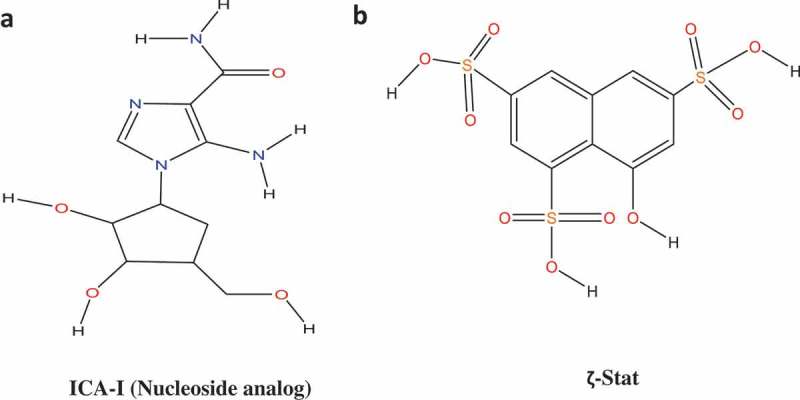
**Structures of Atypical PKC inhibitors**. (**a**) ICA-I (nucleoside analog, 5-amino-1-((1S,2R,3S,4S)-2,3-dihydroxy-4-(hydroxymethyl)cyclopentyl)-1H-imidazole-4-carboxamide). (**b**) ζ-Stat (8-hydroxynaphthalene-1,3,6-trisulfonic acid).

In this study, we used two novel inhibitors of atypical PKC: ICA-I and ζ-Stat (Figure 1), selective for PKC-ι and PKC-ζ respectively, to examine the metastatic behavior of CRC cells. We established that the atypical PKC modulates actin cytoskeleton in colorectal cancer cells by regulating Cofilin via a slingshot isoform, SSH2. Upon PKC-ι and PKC-ζ knockdown, there was an increased expression of p-Cofilin (S3) which was directly correlated to the metastatic behavior of the cancerous cells. Our data indicate that the inhibition of atypical PKC will be a robust therapeutic approach as it disrupts the actin dynamics of the CRC cells.

## Materials and method

### Antibodies and reagents

ICA-I and ζ-Stat were obtained from United Chem Resource (Birmingham, AL) and the National Cancer Institute (NCI) respectively. Anti-PKC-ι (610176) and anti-E-Cadherin (619494) antibodies were purchased from BD Biosciences. The antibodies to anti-Phospho PKC-ζ (T410) (2060), anti-PKC-ζ (9372), anti-SSH1 (13578), anti-Caspase-3 (9662), anti-Cleaved Caspase-3 (9579), anti-PARP (9532), anti- Cleaved PARP (5625), anti-BCL-2 (2872), anti-BCL-XL (2764), anti-ARP2 (5614), anti-α-Tubulin (2125), and anti-GAPDH (5174) were procured from Cell Signaling Technology. The antibodies obtained from Santa Cruz Biotechnology were anti-Cofilin (sc-53934), anti-Phospho Cofilin (S3) (sc-271921), anti-Survivin (sc-419153). Anti-Phospho PKC-ι (T555) (ab5813), anti-Phospho PKC-ζ (T560)(ab59412), and Phalloidin iFluor 594 conjugate (ab176757) were purchased from Abcam. The anti-SSH2 (20674–1-AP) antibody was obtained from ProteinTech. The anti-Phospho SSH (S978)(SP3901) antibody was purchased from ECM Biosciences. The G-actin/F-actin *in-vivo* assay Biochem kit (BK037) was purchased from Cytoskeleton Inc. The siRNAs, siPRKCI (SR321426), and siPRKCZ (SR321432) were procured from Origene. The cell dissociation solution, coating buffer and basement membrane extract (BME) were obtained from Trevigen Inc. The HyQtase cell detachment solution (SV3003001) was procured from Hyclone Inc. Calcein AM (C3100MP) was obtained from Molecular Probes. Enhanced Chemiluminescence (Super Signal West Pico Chemiluminescent Substrate) (34580) was Purchased from Pierce. Horseradish peroxidase (HRP) conjugated goat anti-mouse (1706516), and goat anti-rabbit (1706515) secondary antibodies were bought from Bio-Rad Laboratories. Water-soluble tetrazolium salts (WST-1) (11644807001) reagent was bought from Sigma-Aldrich. Eagle’s minimum essential medium was obtained from Corning. Anti-β-actin (MA5-15739-HRP) antibody, F12K media, and Trypsin-EDTA (ethylene diamine tetra-acetic acid) were purchased from Thermo Fisher Scientific.

### Cell lines and subculture

The healthy colorectal epithelial cells, CCD18CO, and metastatic CRC cell lines, LoVo and RKO, were obtained from American Type Tissue Culture Collection (ATCC). The CCD18CO and RKO cells were sub-cultured and maintained in Eagle’s Minimum Essential Medium (EMEM), and LoVo was sub-cultured and maintained in F12K media. All the flasks were supplemented with 10% Fetal Bovine Serum (FBS) and 1% antibiotics (Penicillin 10 U/ml and streptomycin 10 mg/ml). Cells were incubated at 37°C and 5% CO_2_. Cells were used for the experiments a few days following subculture at 70–80% confluent.

### In-vitro treatment of normal colon and metastatic CRC cells with ICA-I and ζ-stat

The setup for this analysis was the same as our previously published study [28].

### Cell lysates preparation and immunoblot analysis

The experiments were performed as per the experimental procedures described in our previous article [28].

### Transwell invasion and migration assay

After starving for 24 hours, cells were detached from the flask’s surface using cell detachment solution and re-suspended in serum-free media followed by plating (2.5 × 10^4^) into the upper chamber of 96 wells Transwell permeable support (pore size: 8 μm) that has been coated with 0.3x Basement Membrane Extract (BME). Serum containing media was loaded into the receiver plate (lower chamber) as a chemoattractant. LoVo and RKO cells at the upper chamber were treated with 7µM of either ICA-I or ζ-Stat for six days and three days respectively. Following treatment, the invasive cells at the lower chamber were stained with Calcein AM, a fluorescent dye, and quantified using Bio-Tek microplate reader (Winooski, VT) at excitation and emission wavelengths of 485/520 nm.

For migration assay using transwell plate, the same procedure of invasion study was followed, but the transwell inserts were not coated with BME solution.

### Scratch wound healing assay

This assay is performed following the experimental design as our previous work [28].

### Crystal violet staining

Cells were serum starved for 24 hours, followed by detachment and plating (2.5 × 10^4^) into the upper chamber of 96 wells Transwell permeable support (pore size: 8 μm) coated with and without 0.3x Basement Membrane Extract (BME) for studying migration and invasion respectively. Serum (10%) containing media was loaded into the receiver plate (lower chamber) as a chemoattractant. LoVo and RKO cells at the upper chamber were treated with 7µM of either ICA-I or ζ-Stat for six days or three days respectively. The invasive cells in the lower chamber were then fixed with 4% paraformaldehyde, stained with 1% crystal violet in 2% ethanol, washed with water and photographs were captured after drying.

### Phalloidin staining of filamentous (F) actin

CRC cells were grown in 2-wells chamber slides coated with poly D-lysine (1 mg/ml). Following treatment for three consecutive days with 7 μM of either ICA-I or ζ-Stat, cells were fixed with 4% paraformaldehyde. F-actin was subsequently stained with 1X Phalloidin-iFluor 594 in 1% bovine serum albumin (BSA)-phosphate buffered saline (PBS) solution for an hour at room temperature. Cells were washed, counterstained with DAPI and examined under Nikon MICROPHOT-FX fluorescence microscope (Ex/Em = 590/618) and photographs were captured using ProgRes®Capture 2.9.0.1.

### 4΄,6-diamidino-2-phenylindole (DAPI) staining

The procedures followed was identical as in our previously published study [28].

### Filamentous (F) and globular (G) actin fractionation

Fractionation of F-actin and G-actin was performed according to manufacturer instruction with G-Actin/F-actin *in-vivo* assay kit. Briefly, CRC cells were grown in 100 mm tissue culture plate and treated with 7 μM of either ICA-I or ζ-Stat for three consecutive days. Cells were then lysed with cell lysis buffer containing F-actin stabilizing buffer to extract G-actin, followed by extraction of F-actin. The F-actin was then depolymerized using an F-actin depolymerizing buffer to convert F-actin to G-actin. F/G fractions were resolved using the 10% SDS-PAGE and immunoblotted using Actin antibody. The intensity of bands from different fractions was then quantified by densitometry.

### Transfection of metastatic CRC cells and RNA interference

Approximately, 1 × 10^6^ CRC cells were inoculated into 60 mm tissue culture plate. Twenty-four hours post-plating, cells were transfected using Si Tran 1.0 (Origene, Rockville, MD) according to the manufacturer instructions. The siPRKCI (sequence 5ʹ-UUAUGAGCUAAACAAGGAUUCUGAA-3ʹ) and siPRKCZ (sequence 5ʹ-AGUAGAGCACAAGAACGAGGACGCC-3ʹ) were used to transfect the LoVo and RKO cells. Additionally, cells were also transfected with universal scrambled negative control sequence to ensure the targeted gene silencing. Following forty-eight hours of incubation, cells were harvested and subjected to immunoblot analysis to determine the expression of PKC-ι, PKC-ζ, SSH1, SSH2, Cofilin and pCofilin (S3).

### Densitometry

The intensity of each band was quantified using 1D analysis software, Alpha View (Protein Simple, San Jose, California) and Image Studio Lite Ver 5.2 (LI-COR Biosciences, Lincoln, NE). The background intensity was subtracted from each band to quantify the correct intensity.

### Statistical analysis

To determine the Statistical significance of the data, the results were expressed as the Mean ± Standard error of the mean (S.E.M) of at least three independent experiments. *p* values were calculated based on Student t-test (two-tailed) and one-way ANOVA using GraphPad Prism 7.04 or Microsoft Excel software. A *p* value of < 0.05 was considered significant.

## Results

### Expression profile of atypical PKC in rapidly growing and serum starved healthy and malignant colon cells

**Figure 2.**
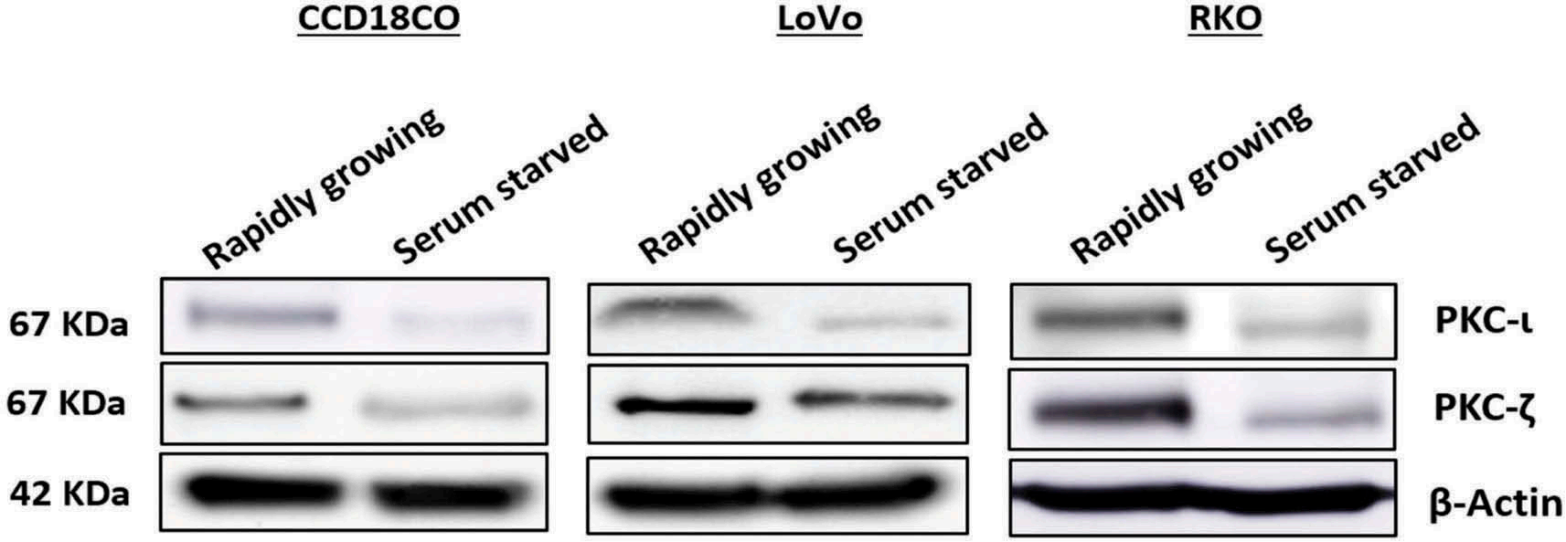
**Expression profile of atypical PKCs in normal and metastatic CRC cells**. Cells were grown with and without serum over a period of 48 hours. Whole cell lysate was prepared and an equal amount of protein from the lysates was subjected to western blot analysis, followed by examining PKC-ι and PKC-ζ expression in rapidly growing and serum starved cells. β-Actin was used as a loading control. *N *= at least 3 independent experiments.

To establish the role of atypical PKC in colorectal cancer the very first experiment performed was the determination of atypical PKC expression profile in rapidly growing and serum starved slow growing normal and colorectal cancerous cells. Our data indicated that the expression of PKC-ζ was higher in rapidly growing CRC cells (both LoVo and RKO) compared to the healthy cell (CCD18CO). Additionally, PKC-ι was also found to be elevated in fast-growing metastatic RKO cell. However, the expression profile of atypical PKC in the normal cell was not as robust as in cancerous cells (Figure 2). These results suggest that the atypical PKC might play an essential role in CRC cells progression.

### ICA-I and ζ-stat inhibit PKC-ι and PKC-ζ respectively

**Figure 3.**
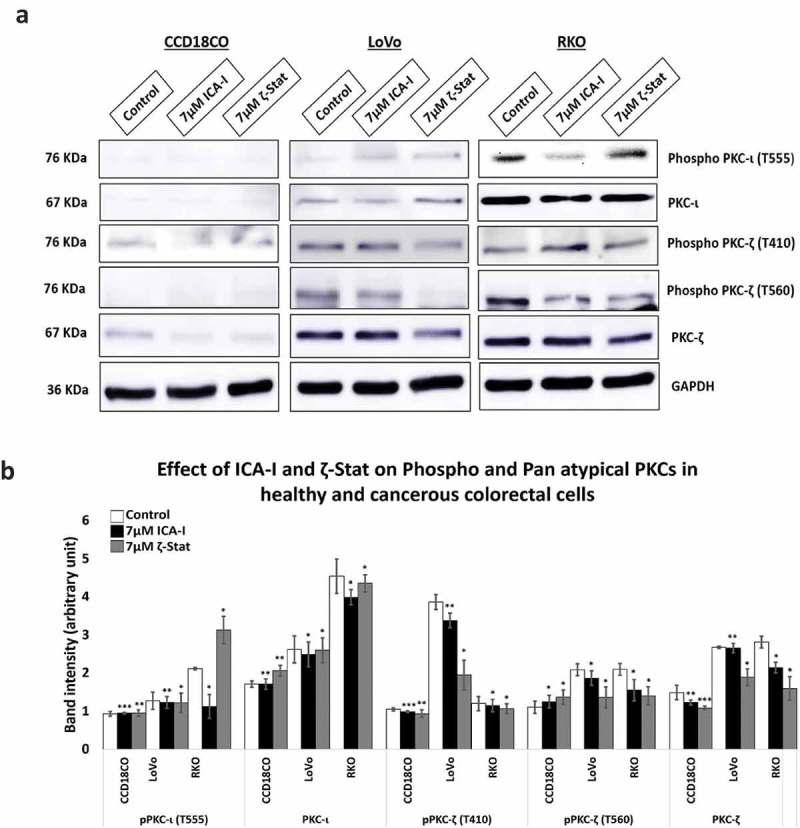
**atypical PKC inhibitors reduced the expression of both phospho and pan level of atypical PKCs**. (**a**) Western blot analysis for the effect of ICA-I and ζ-Stat on PKC-ι and PKC-ζ in CCD18CO, LoVo and RKO cells following three days of treatment. (**b**) The bar graphs illustrate densitometry of total and phospho atypical PKC proteins as a function of atypical PKC inhibitors. Cells were grown in 100 mm cell culture plate followed by treatment with 7 μM of either ICA-I or ζ-Stat. Mean ± S.E.M.; *N *= at least 3 separate experiment; * = 0.05, ** = 0.02, *** = 0.01(indicates *p* value).

The amino acids sequences of PKC-ι and PKC-ζ are highly identical (72% homologous) [17]. Hence, the determination of selectivity of the inhibitors used was necessary. Therefore, cells (both normal and malignant) were treated with 7 μM of either ICA-I or ζ-Stat and examined for the expression of atypical PKC. Our data revealed that with the treatment of ζ-Stat, the level of both phospho and Pan PKC- ζ decreased by more than 30% (*p* < 0.05) in CRC cells. Additionally, ICA-I brought a significant reduction in the Phospho PKC-ι (T555) by more than 45% (*p* < 0.05) in RKO cells but not in LoVo. In contrast, the inhibitors did not induce any notable change in the atypical PKC levels of the healthy cell (Figure 3(a,b)). These observations indicate that the inhibitors are specific for the respective atypical PKC in CRC cells and their inhibition could be used as a therapeutic target to encounter colorectal cancer progression.

### Atypical PKC regulates CRC cells migration and invasion

Our previous study showed that PKC-ζ regulates the growth of colorectal cancers cells via PKC-ζ/Rac1/Pak1/β-Catenin pathway [28,29]. Our next target was to examine the role of atypical PKC in another hallmark of cancer, metastasis. To gain insight into the anti-migratory and anti-invasive potential of atypical PKC inhibitors, we performed three different strategies: scratch wound healing assay, transwell migration-invasion assay and crystal violet staining of the cells passed through BME. The data from scratch assay revealed that the treatment (six days) of LoVo with ζ-Stat (at 7 μM) decreased the rate of wound closure by approximately 45% (*p*< 0.05) compared to control. The treatment (three days) of RKO with both atypical PKC inhibitors (ICA-I and ζ-Stat, at 7 μM) showed a prolonged rate of wound closure (approximately 35%, *p*< 0.02) compared to control. However, treatment with ICA-I did not bring any significant effect in LoVo (Figure 4(a)). Similarly, the transwell migration and invasion assay results showed that atypical PKC knockdown significantly inhibited the chemotactic migration of LoVo (approximately 40%, p < 0.05) and RKO (approximately 40%, p < 0.01) CRC cells (Figure 4(b)). According to our transwell invasion and crystal violet staining assays, the treatment (at 7 μM) of both ICA-I and ζ-Stat decreased the number of RKO cells that invaded through the BME (approximately 45%, *p* < 0.01), likewise, in response to ζ-Stat, the number of LoVo cells passed into the lower chamber of transwell plate through the BME reduced remarkably (about 50%, *p* < 0.02) (Figure 4(c)). E-Cadherin, a cell adhesion molecule, is often downregulated and considered to be a crucial mediator of epithelial to mesenchymal transition (EMT) during metastasis [30]. Additionally, there was an elevated expression of E-Cadherin, as a function of PKC-ζ inhibition in LoVo, and PKC-ι and PKC-ζ inhibition in RKO (Figure 4(d,e)). These data indicate that atypical PKC is a crucial regulator in CRC migration and invasion.

**Figure 4.**
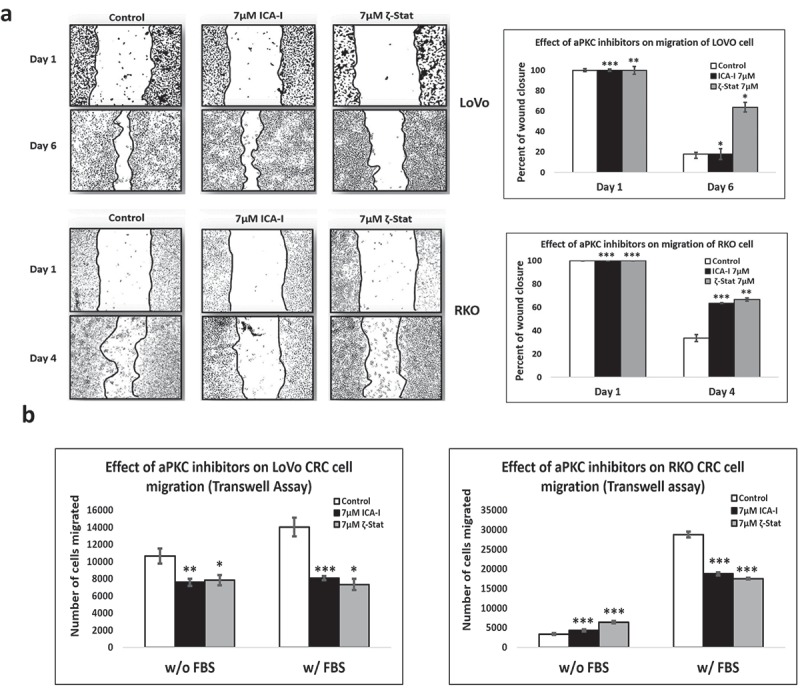
**Atypical PKCs regulate migration and invasion of colorectal cancer cells**. (a) Metastatic LoVo and RKO cells were grown to approximately 90% confluency and subjected to wound healing assay by making a scratch using sterile 100 µl pipette tip along with atypical PKC inhibition treatment. (b) The bar charts are representing the number of cells migrated in transwell plate against chemotactic gradient. (c) Crystal violet stained cells, and bar charts are showing the number of CRC cells passed through BME into the lower chamber of transwell plate. (d) Expression of E-cadherin, a cell adhesion protein, in treated healthy and CRC cells. (e) Densitometry of E-cadherin expression. All the experiments were performed at least three times. Mean ± S.E.M. * represents *p* value (* < 0.05, ** < 0.02, *** < 0.01).

**Figure 4.**
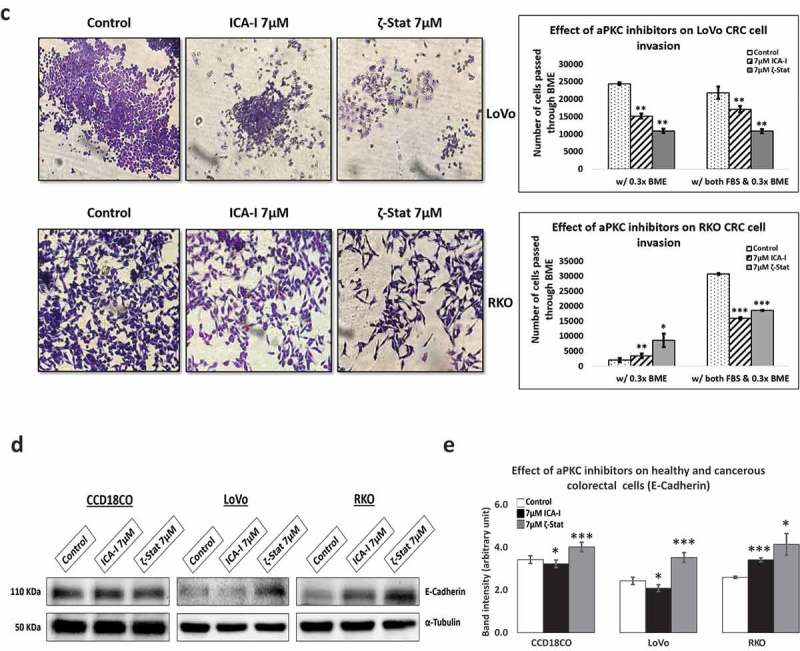
(Continued)

### Atypical PKC controls cofilin/p-cofilin ratio via slingshot

**Figure 5.**
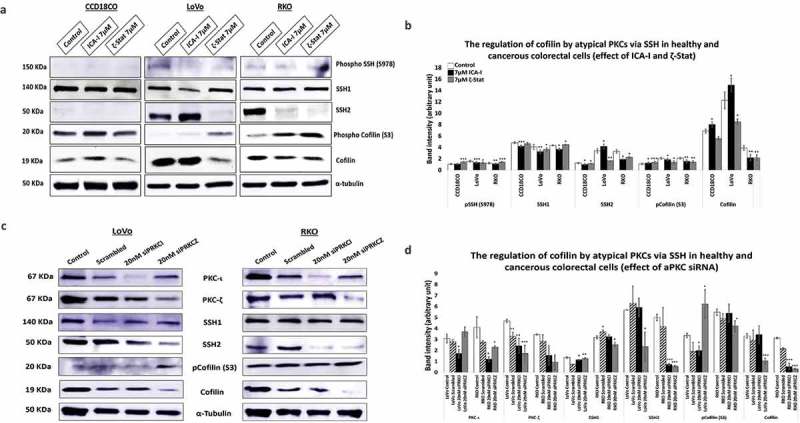
**Atypical PKC controls actin organization by regulating Cofilin via SSH2 in CRC cells**. (**a**) Immunoblot analysis is showing the effect of ICA-I and ζ-Stat in healthy and CRC cells following three days of treatment. Proteins tested were pSSH (S978), SSH1, SSH2, pCofilin (S3) and Cofilin. (**c**) The expression of PKC-ι, PKC-ζ, SSH1, SSH2, pCofilin (S3) and Cofilin in si*PRKCI* and si*PRKCZ* transfected CRC cells. (**B & D**) The bar charts are representing the corresponding band intensities. α-tubulin was probed as a loading control. All experiments were performed for at least 3 independent times. Mean ± S.E.M and * represents *p* value (* < 0.05, ** < 0.02, *** < 0.01).

Our next target was to study possible protein/s that drives the changes in the cytoskeletal phenotype of CRC cells. Since actin filaments are the major components of cell cytoskeleton, we decided to test the essential actin binding candidate namely Cofilin as it severs F-actin to promote polymerization. Our results showed that the treatment of ζ-Stat reduced the amount of total Cofilin by more than 30% (*p* < 0.05) and increased the Phospho Cofilin at S3 by 50% (*p* < 0.05) in both CRC cells. Additionally, ICA-I showed the similar shift in RKO but not in LoVo (Figure 5(a,b)). Slingshot proteins are significant phosphatases that act on Cofilin activity [31]. We established that the phosphatase SSH2 levels in LoVo and RKO decreased dramatically (50% in LoVo, *p* < 0.02; and 30% in RKO, *p* < 0.05) with 7 μM ζ-Stat, however, the phospho SSH at 978 and SSH1 levels did not change significantly. Similarly, 7 μM ICA-I decreased the SSH2 expression by 42% (*p* < 0.05) in RKO but not in LoVo. Conversely, CCD18CO healthy colon cells were insensitive to atypical PKC knockdown treatment (Figure 5A, 5B). As a proof of concept, we also tested the expression of PKC-ι, PKC-ζ, SSH1, SSH2, pCofilin(S3) and Cofilin in si*PRKCI* and si*PRKCZ* transfected CRC cells. Our findings illustrated that the si*PRKCI* and si*PRKCZ* transfected CRC cells showed a similar trend in the expression of targeted proteins as in cells treated with ICA-I and ζ-Stat (Figure 5(c,d)). These data suggest that atypical PKC stimulates actin remodeling in CRC cells by regulating Cofilin via SSH2.

### Atypical PKC controls filamentous actin and actin dynamics

**Figure 6.**
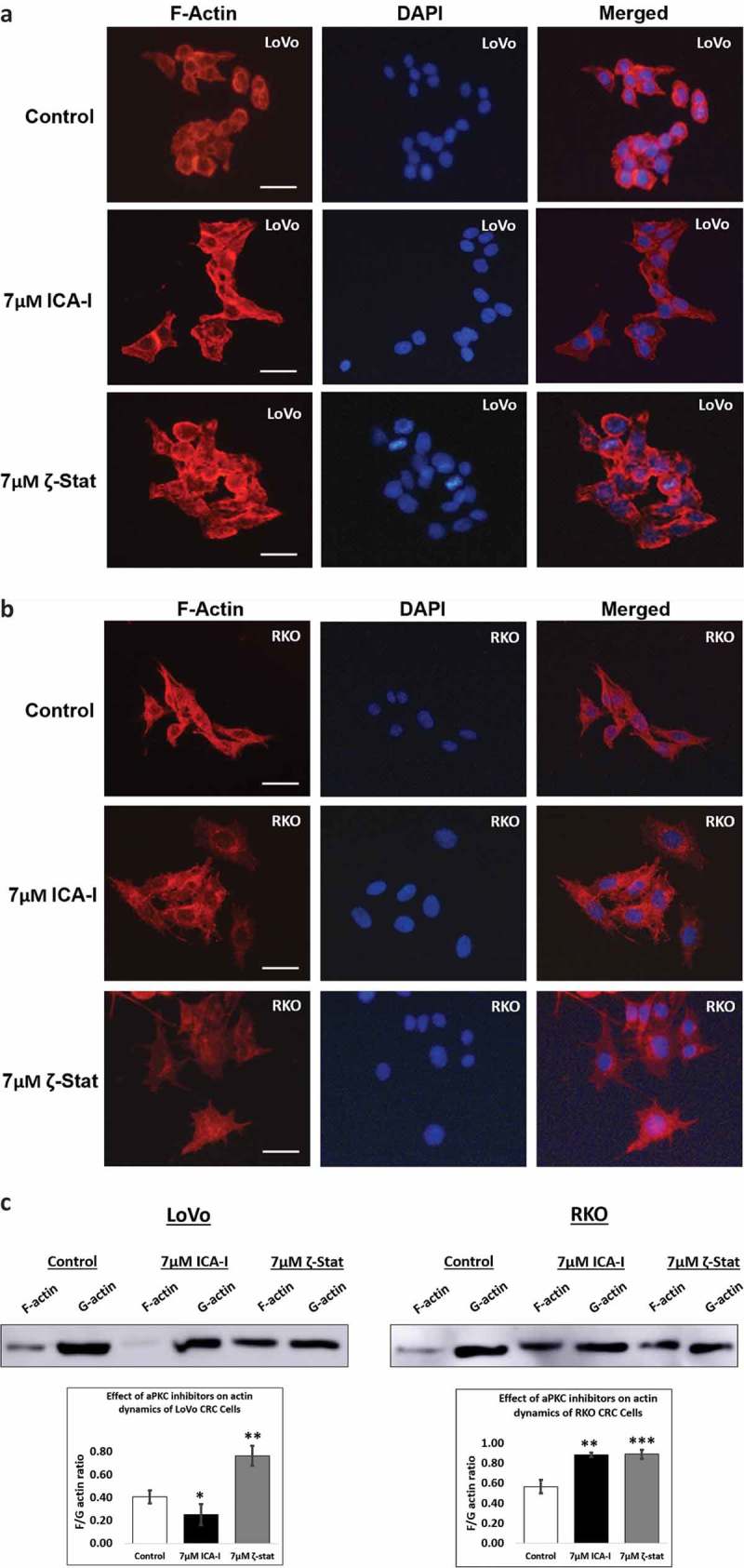
**Atypical PKC regulates filamentous actin content and actin dynamics**. Cells were treated with atypical PKC inhibitors for 72 hours. (**A, B**) Cells were stained with Phalloidin iFluor 594 and counter stained with DAPI. Original magnification: 20X and scale bars represent 10 μm. (**c**) F-actin and G-actin were fractionated from control and treated cells followed by western blot analysis. The F and G-actin were quantified using densitometry followed by determining F/G actin ratio. (Mean ± S.E.M, *N *= at least 3 independent experiments, * indicates *p* value where * < 0.05, ** < 0.02, *** < 0.01).

During chemotaxis, two crucial events are cytoskeleton rearrangement and adhesion which are regulated by PKC-ζ [26]. To investigate how spreading and metastasis of the CRC cells are affected by atypical PKC, actin filaments were examined under the microscope with phalloidin conjugate following atypical PKC inhibition. Down-regulation of PKC-ζ provoked excellent actin filaments organization in both CRC cells compared to control cells that contained less organized actin filament (Figure 6(a,b)). Likewise, ICA-I significantly changed the actin filaments organization in RKO cell as well (Figure 6(b)). After observing changes in cells migratory behavior and actin filaments, we also decided to study actin dynamics. Hence, we determined the fractions of F-actin and G-actin in treated and untreated LoVo ad RKO cells. In response to PKC-ζ knockdown, the ratio of F/G actin increased significantly (approximately 35%, *p* < 0.02) in both CRC cells (Figure 6(c)). Additionally, inhibition of PKC-ι increased the F/G ratio by 31% (p < 0.02) in RKO cell too (Figure 6(c)). These results suggest that atypical PKC facilitates metastasis of CRC cells by modulating actin dynamics.

### Atypical PKC inhibition prevents the dendritic branches by blocking ARP2

**Figure 7.**
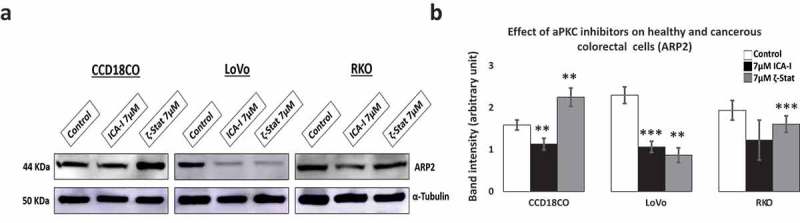
**Expression of actin related protein 2, ARP2 in normal and CRC cells**. Cells were grown and treated with atypical PKC inhibitors for three consecutive days. Equal amount of cell lysate was subjected to western blot analysis. (**a**) Expression of ARP2, an actin nucleating protein in healthy and cancerous cells. α-Tubulin was used as a loading control. (**b**) The bar charts represent corresponding band intensity. *N* = 3 independent experiments. Mean ± S.E.M. * represents *p* value (* < 0.05, ** < 0.02, *** < 0.01).

Actin-related protein 2, ARP2, resembles monomeric actin and coordinately overexpressed with Cofilin in motile cells [32]. ARP2 synergistically interacts with Cofilin to produce dendritic nucleation which pushes the cell membrane inducing cell protrusion and lamellipodia extension [33]. To establish the fate of branching structures of actin filaments in atypical PKC knockdown cells, we examined the expression of ARP2 in ICA-I and ζ-Stat treated cells. Our data showed that the expression ARP2 decreased by more than 50% (*p* < 0.02) in LoVo with the inhibition of either PKC-ι or PKC-ζ. Additionally, ICA-I brought a significant reduction (approx. 35%) of ARP2 levels in RKO cell (Figure 7(a,b)). These results indicate that the atypical PKC may also regulate dendritic nucleation in motile CRC cells.

### Atypical PKC inhibitors hinder CRC cells viability without affecting normal colon cells

**Figure 8.**
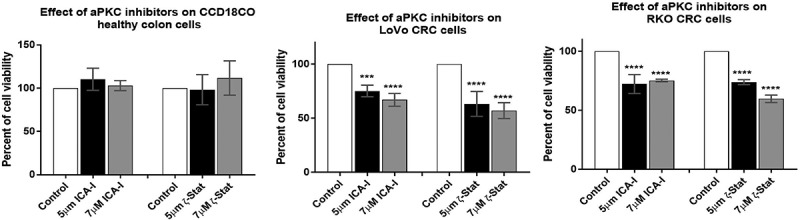
**Effect of atypical PKC inhibitors on normal and malignant colorectal cells**. CCD18CO, LoVo, and RKO cells (4 x10^3^) were plated in 96 wells plate and treated with 5 μM and 7 μM of either ICA-I or ζ-Stat for 72 hours followed by incubation with a WST-1 reagent for 3 hours and determination of absorbance at 450 nm using microplate reader. The data represents *N *= at least 3 independent experiments, mean ± S.E.M. *** < 0.0009, and **** < 0.0001 (indicate *p* value).

To determine the effect of atypical PKC on colorectal cell proliferation, both normal and cancerous cells were treated with either 5 μM or 7 μM of either of the two inhibitors (ICA-I and ζ-Stat) for three consecutive days. The results showed that ζ-Stat reduced the viability of cancerous cells (both LoVo and RKO) by approximately 45% at a dose of 7 μM (*p*< 0.0001) (Figure 8). Likewise, the treatment of 7 μM ICA-I decreased the cells viability of LoVo by 33% (*p*< 0.0009) and RKO by 46% (*p*< 0.0001) (Figure 8). However, the treatment with atypical PKC inhibitors did not produce any significant cytotoxic effect on normal colon cell viability. These data demonstrate that the atypical PKC inhibitors might be used to treat CRC cells without affecting healthy colorectal cells.

### Induction of apoptosis by atypical PKC inhibitors in CRC cells

**Figure 9.**
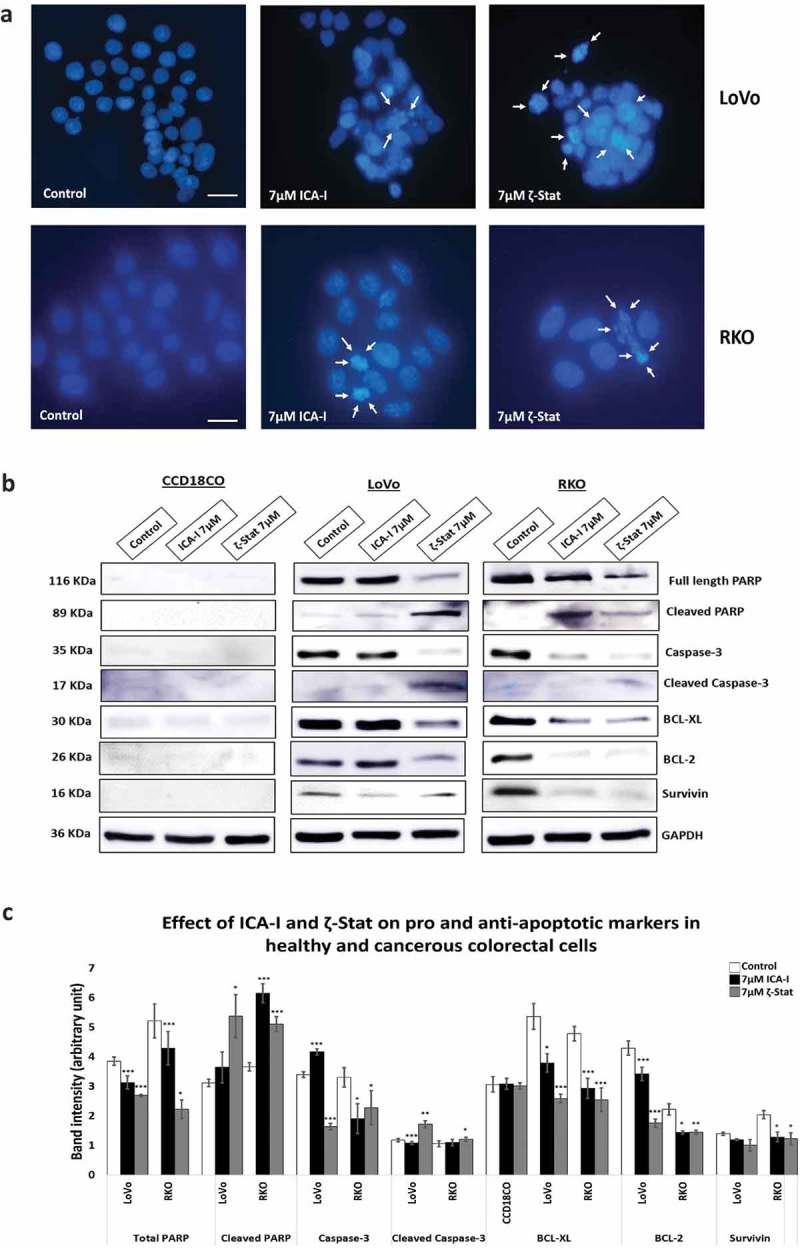
**Atypical PKC inhibitors induce apoptosis in CRC cells**. Cells were treated for 72 hours with 7 μM of either ICA-I or ζ-Stat. Following treatment, (**a**) Cellular nuclei were visualized using DAPI. Original magnification 20X and scale bars represent 10 μm. The white arrows are showing the cells undergoing apoptosis. (**b**) An equal amount of protein (40 μg) from cellular extracts of both normal and CRC cells were separated by SDS-PAGE, followed by Western blot analysis with different pro and anti-apoptotic antibodies. GAPDH was used as a loading control. (**c**) The bar charts represent the corresponding band intensities of apoptotic and survival markers. *N *= 3, Mean ± S.E.M. * represents *p* value where * < 0.05, ** < 0.02 and *** < 0.01.

To establish whether the cells were undergoing programmed cell death with atypical PKC knockdown, visualization of cellular nuclei using DAPI along with the determination of expression of different pro- and anti-survival proteins were investigated. The data showed that with an ICA-I treatment of 7 μM for 72 hours, RKO cells exhibited notable chromatin condensation and nuclear fragmentation compared to control as visualized by nuclear staining with DAPI, likewise, to some extent, LoVo also possessed fragmented cells (Figure 9(a)). On the other hand, in response to ζ-Stat (7 μM) treatment, both LoVo and RKO cells showed significant apoptotic bodies. Additionally, PKC-ζ inhibition reduced the level of survival proteins, such as BCL-2 by more than 35% (*p* < 0.02), BCL-XL by more than 45% (*p* < 0.01), and Survivin by more than 30% in both CRC cells (Figure 9(b,c)). Similarly, PKC-ι inhibition brought down the level of BCL-2, BCL-XL, and Survivin by more than 35% (*p* < 0.05) in RKO but not in LoVo. Moreover, Caspase-3 was found to be active as proportional changes in the expression of Cleaved and total Caspase-3 were observed. Furthermore, changes in the expression of PARP, a downstream nuclear substrate of Caspase-3 activation [34], was also detected (Figure 9(b,c)). However, no notable apoptotic changes were observed in normal colorectal cells with atypical PKC inhibition. Hence, these findings indicate that the CRC cells undergo apoptosis in response to anti-atypical PKC treatment.

## Discussion

Our current understanding concerning PKCs recalls that they are functionally pleiotropic protein kinases involved in various parallel and cross signaling events that regulate different cellular processes such as proliferation and metastasis [35]. During carcinogenesis, PKCs are active and found to be elevated in many cancers, such as breast, lung, liver, colon, and prostate [36]. Most importantly, each isoform of PKC family may act distinctively in the biological system and tumorigenesis. Therefore, careful dissection of PKC function and tailored intervention strategy in cancer progression need to be established.

Colorectal adenocarcinoma is cancer that originates from glands found in the wall of the colon and rectum and accounts for 96% of CRC [37]. Thus, the rationale for choosing LoVo and RKO in this study was that both cells represent colorectal adenocarcinoma. Atypical PKC is highly expressed in more than 120 colorectal adenocarcinoma tissue samples [38], likewise, in this study, PKC-ζ is overexpressed in both CRC cells and PKC-ι in RKO cells (Figure 2). The selectivity of the inhibitors in different CRC cell lines was necessary because of high structural similarity (72% identical) of atypical PKC isoforms. Our data illustrated that ICA-I and ζ-Stat inhibited PKC-ι and PKC-ζ, respectively (Figure 3(a,b)).

Previously, our published study showed that the PKC-ζ regulate the growth and proliferation of CRC cells by stimulating Rac1/Pak1/β-Catenin pathway [28,29]. In this study, we evaluated the functional role of atypical PKC in the metastasis of CRC cells. Firstly, we performed two primary metastasis assays, namely scratch wound healing assay and serum gradient induced chemotaxis assay, using metastatic LoVo and RKO cells. In each case, the treatment of atypical PKC inhibitors impaired the migration and invasion of the CRC cells. Briefly, PKC-ζ inhibition blocked the metastasis of both CRC cells and PKC-ι knockdown brought a significant reduction of migration and invasion of RKO as indicated by our scratch wound healing assay, crystal violet staining and transwell migration-invasion assay (Figure 4(a-c)). In LoVo, even though the knockdown of PKC-ι did not bring any significant change in the scratch wound recovery assay (Figure 4(a)), a notable change was observed in the serum gradient induced chemotaxis assay with the treatment of ICA-I (Figure 4(b,c)). The exact reason for this finding could not be deciphered. However, the inhibition of PKC-ζ induced the upregulation of E-Cadherin in both CRC cells, whereas, only PKC-ι knockdown promoted the increased E-Cadherin levels in LoVo (Figure 4(d,e)). Furthermore, our subsequent metastatic investigation of LoVo showed that only PKC-ζ could regulate the metastasis of LoVo by modulating actin organization.

The crawling movement of metastatic cancerous cells from one place to another is driven by the actin polymerization and depolymerization. Cofilin is an actin-binding protein (ABP) which regulates actin polymerization and the formation of migratory structures of the cells, i.e., lamellipodia and filopodia to promote directional cell movement [12]. Activated Cofilin has been linked with metastatic glioblastoma, breast, pancreatic and ovarian cancer [39–41]. In addition, elevated expression of cofilin is also implicated in CRC cells [42,43]. Moreover, constitutive Cofilin phosphorylation at serine-3 residue retards the ability of CRC cells to metastasize [44]. Hence, we examined the expression of both phospho and pan Cofilin in atypical PKC inhibitors pretreated CRC cells. Our data demonstrated that the inhibition of PKC-ζ reduced the expression of Cofilin in both LoVo and RKO, and the knockdown of PKC-ι decreased Cofilin levels in RKO as well. In addition, treatment of atypical PKC inhibitors increased the cellular level of Phospho-Cofilin (S3) in both CRC cells (Figure 5(a,b)). Thus, our observation supports a possible role of atypical PKC in the migration and invasion of CRC cells by impairing downstream signals that regulate Cofilin.

In CRC, an exciting novel finding was that the phosphatase, SSH, an upstream regulator of Cofilin dephosphorylation, is also overexpressed [45]. Elevated levels of SSH is not only associated with tumorigenesis but also a significant predictor of lymph node metastasis [11,45,46]. In further agreement, *in-vitro* studies of pancreatic and breast cancer cell lines suggest that the tumor-promoting role of SSH is mainly due to its ability to dephosphorylate Cofilin since the loss of SSH function resulted in the decreased non-phosphorylated Cofilin and metastatic ability of those cell lines [31,47]. With this context, we examined two isoforms of SSH, SSH1, and SSH2, in treated and untreated CRC cells as a function of atypical PKC inhibition. Our results depicted that the knockdown of atypical PKC did not change SSH1 levels, but, resulted in the remarkable deactivation of SSH2 in both CRC cell lines which were correlated to the reduced level of activated Cofilin and increased level of phosphorylated Cofilin (Figure 5(a,b)). As a proof of concept, CRC cells were also transfected with siRNA of *PRKCI* and *PRKCZ* (genes for PKC-ι and PKC-ζ respectively). Our observation showed a similar correlation among atypical PKC, SSH2 and Cofilin expression in siRNA transfected CRC cells as in atypical PKC inhibitors treated cells (Figure 5(c,d)).

Since cofilin activity is pivotal for cellular actin filaments polymerization/depolymerization and branching that ultimately leads to membrane protrusion and polarization, this issue was further addressed by immunostaining F-actin and measuring the level of F and G-actin in treated CRC cells. We found that the actin filaments were nicely organized around the cytoskeleton of treated LoVo cell with ζ-Stat and treated RKO cell with both atypical PKC inhibitors (Figure 6(a,b)). Additionally, the ratio of F/G actin was increased notably in both LoVo and RKO cells with the treatment of atypical PKC inhibition (Figure 6(c)). Moreover, another crucial step in invasion and protrusion is the formation of branched dendritic actin network [48]. Cofilin partners with ARP2 to stimulate the formation of dendritic structure [48]. Our data illustrated that the inhibition of atypical PKC in CRC cells reduced the level of ARP2 in treated cancerous cells compared to both healthy colon cells and untreated cancerous cells (Figure 7(a,b)). Moreover, the primary signaling molecules that drive the interaction of Cofilin and ARP2 are small GTPases such as Rho, and Rac1 [49]. Previously, we found that the PKC-ζ associates and phosphorylates Rac1, hence, it may also facilitate the Cofilin-ARP2 interaction as well. Therefore, the data presented here have an established part of the mechanism of CRC cells metastasis regulated by atypical PKC via Cofilin. However, further *in-vitro* and *in-vivo* studies need to be performed to fully elucidate the metastatic role of atypical PKC in the regulation mechanism of Cofilin by slingshot.

Furthermore, we also evaluated the effect of atypical PKC on CRC cells proliferation and apoptosis. The treatment with ICA-I or ζ-Stat significantly reduced the proliferation of CRC cells (Figure 8). Likewise, LoVo and RKO cells were undergoing apoptosis as evident by the visualization of apoptotic bodies (Figure 9(a)) and changes in the expression of apoptotic and survival markers such as BCL-XL, BCL-2, and survivin. The activation of Caspase cascade is a critical event in a cell undergoing apoptosis [50]. PARP, a nuclear enzyme, helps in DNA repair and maintain genomic integrity in healthy cells. Cleaved PARP by active Caspases is a marker of apoptotic cell death [51]. Caspase cascade activation was confirmed by the change in the levels of total and cleaved Caspase-3 and PARP (Figure 9(b,c)). Further investigations need to be performed to decrypt the whole apoptotic mechanisms in atypical PKC inhibitors treated CRC cells.

**Figure 10.**
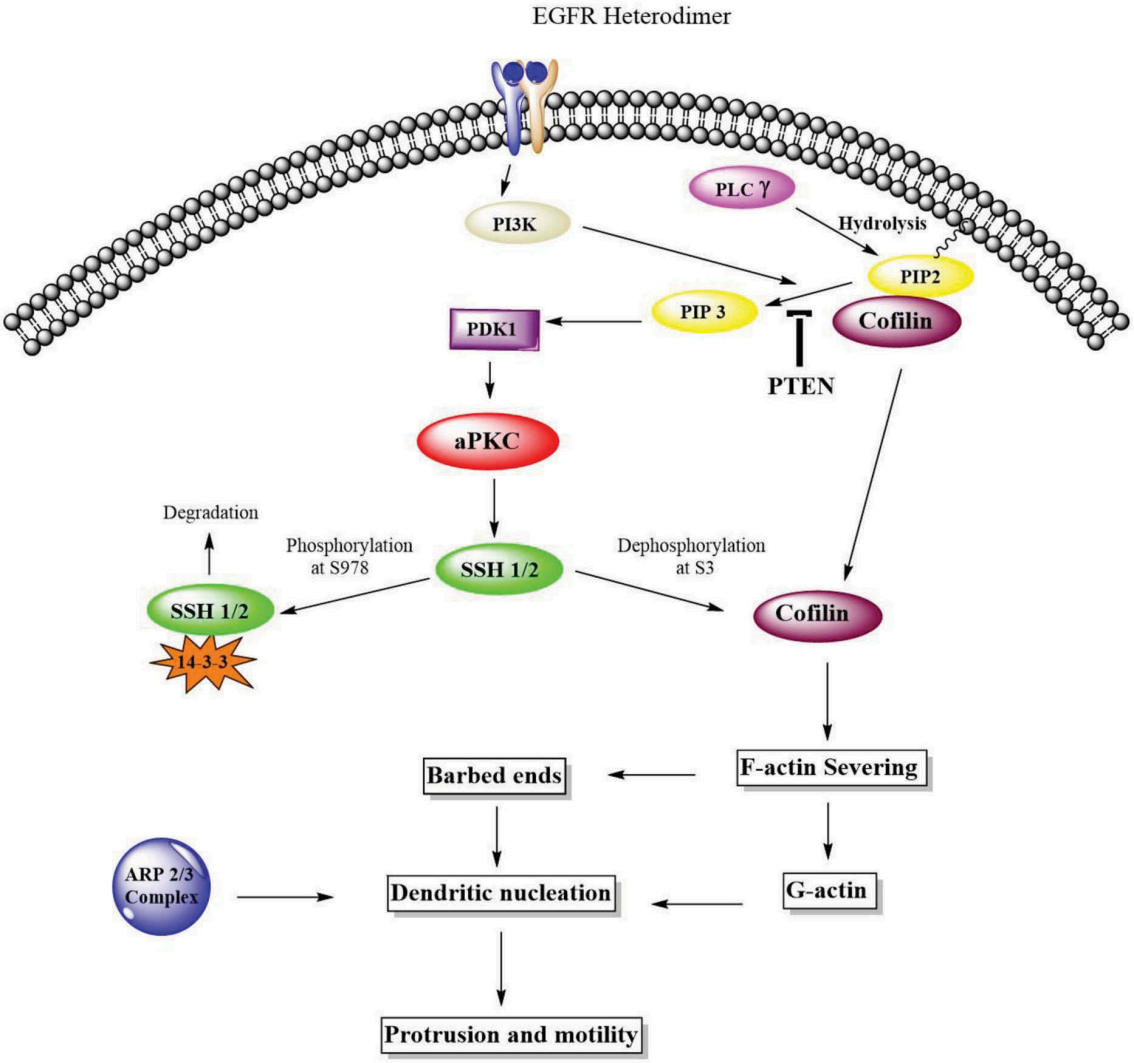
**Atypical PKCs control actin cytoskeleton via Cofilin activation**. The Cofilin pathway is activated by atypical PKC via Slingshot, SSH. Phosphorylated Cofilin at serine-3 is inactive. Phosphatases such as SSH induces the dephosphorylation and activation of Cofilin. Activated Cofilin can then sever the mother filaments and produced free barbed ends leading to the elongation of newly formed actin filaments which are preferred for dendritic nucleation by ARP2/3 complex and globular actin. These newly formed structures push on the cell membrane to promote protrusion and motility.

Collectively, we can conclude that PKC-ζ is elevated in both LoVo and RKO cells, PKC-ι is overexpressed in RKO cell, and the atypical PKC when amplified or mutated are implicated in the metastatic progression of CRC by regulating actin cytoskeleton of CRC cells via Cofilin activation (Figure 10). Furthermore, the expression of atypical PKC should be used as a biomarker for CRC when personalized treatment is sought for anti-atypical PKC therapy.
